# Supplementary material for: The Role of Different Immunocompetent Cell Populations in the Pathogenesis of Head and Neck Cancer—Regulatory Mechanisms of Pro- and Anti-Cancer Activity and Their Impact on Immunotherapy
Source: Cancers (Basel). 2023 Mar 7;15(6):1642. doi: 10.3390/cancers15061642 (PMC10046400; doi:10.3390/cancers15061642)
Supplement: Supplementary file 1 [file cancers-15-01642-s001.zip › cancers-2236578-supplementary.pdf]

Supplementary Table S1. Selected clinical trials on the use of immunotherapy incorporated ICIs in the HNSCC treatment strategy.

Treatment settings (Trial; phase)	Specification and description	Objective	Results
ICIs + CHT			
KEYNOTE-048 (NCT02358031) Phase 3	Pembrolizumab (a highly selective humanized monoclonal antibody that directly blocks the interaction of PD-1 and each of its ligands) monotherapy vs. Pembrolizumab + platinum-based CT (Cisplatin or Carboplatin) + 5-Fluorouracil (5-FU) vs. Cetuximab + platinum-based CT (Cisplatin or Carboplatin) + 5-FU.	Pembrolizumab as 1st line treatment of R/M HNSCC<br>Primary end point is progression-free survival per RECIST 1.1 as assessed by central radiologist review. Secondary end points: overall survival, proportion progression free at 6 and 12 months, overall response rate, and response duration.	Pembrolizumab alone improved OS over SOC in the PD-L1 CPS ≥20 ($p = 0.0007$) and ≥ 1 ($p = 0.0086$) populations.<br>Pembrolizumab + CT significantly improved OS in the total population ($p = 0.0034$).
CheckMate 141 (NCT02105636) Phase 3	Nivolumab (human IgG4 monoclonal antibody to the programmed death-1, PD-1 receptor) monotherapy vs. SOC (Cetuximab + Cisplatin/ Carboplatin + Fluorouracil) (Extreme Study Regimen)	The primary end point was overall survival. Additional end points included progression-free survival, rate of objective response, safety, and patient-reported quality of life.	Among patients with platinum-refractory, recurrent squamous-cell carcinoma of the head and neck, treatment with Nivolumab resulted in longer overall survival than treatment with standard, single-agent therapy.
KEYNOTE-040 (NCT02252042) Phase 3	Pembrolizumab (a highly selective humanized monoclonal antibody that directly blocks the interaction of PD-1 and each of its ligands) monotherapy vs. SOC (Cetuximab + Cisplatin/ Carboplatin + Fluorouracil) (Extreme Study Regimen)	A phase III randomized trial of pembrolizumab (MK-3475) versus standard treatment in patients with recurrent or metastatic head and neck cancer.<br>Primary end points are progression free survival (PFS) and overall survival (OS); secondary end points include ORR, DOR and PFS, OS, and ORR in PD-L1+ patients. Treatment differences in PFS and OS will be assessed using stratified log-rank test; Hazard ratios with 95% confidence intervals will be estimated using stratified Cox proportional hazard models.	The clinically meaningful prolongation of overall survival and favourable safety profile of Pembrolizumab in patients with recurrent or metastatic head and neck squamous cell carcinoma support the further evaluation of Pembrolizumab as a monotherapy and as part of combination therapy in earlier stages of disease.<br>Fewer patients treated with Pembrolizumab than with standard of care had grade 3 or worse treatment-related adverse events.
ICIs dual checkpoint blockade			
EAGLE (NCT02369874) Phase 3	Durvalumab (anti-PD-L1 mAb) monotherapy vs. Durvalumab + Tremelimumab (anti-CTLA-4 mAb) vs. SOC in R/M. Eligible patients are immunotherapy naïve but have progressed on a platinum-containing regimen or within 6 months of multimodality platinum therapy.	2nd line treatment for R/M HNSCC targeting both PD-1 and CTLA-4 pathways may induce synergistic antitumor effects.<br>The primary endpoint was overall survival (OS) with dual primary objectives of D+T vs SOC and D vs SOC. Additional endpoints included objective response rate (ORR), duration of response (DoR), and adverse events (AEs).	Failed to meet primary endpoint of improved overall survival.<br>D and D+T did not demonstrate a statistically significant improvement in OS compared to standard chemotherapy in pts with R/M HNSCC. Median OS and ORR of D arm were similar to other studies with checkpoint inhibitors. The SOC arm outperformed what has been seen for SOC arms in previous studies; subsequent immunotherapy may have confounded the OS analyses. The safety profile for D and D + T in R/M HNSCC is consistent with previous trials.
CheckMate 651 (NCT02741570) Phase 3	Nivolumab (human IgG4 monoclonal antibody to the programmed death-1, PD-1) receptor + Ipilimumab (ipi; a humanized IgG1 CTLA-4 checkpoint inhibitor) vs. SOC (Cetuximab + Cisplatin/Carboplatin + Fluorouracil) (Extreme Study	Combination Nivolumab + ipilimumab has shown significant promise in patients with NSCLC, advanced melanoma and advanced RCC.<br>Primary endpoints are OS and progression-free survival. Secondary endpoints are objective response rate,	Results ongoing. Primary outcome measures include OS and PFS in patients with PD-L1 expressing tumours.

	Regimen) as first-line treatment in patients with R/M HNSCC.	time to symptomatic deterioration based on the 10-item Functional Assessment of Cancer Therapy-Head & Neck Symptom Index, and PD-L1 expression as a predictive biomarker for efficacy.	
KESTREL (NCT02551159) Phase 3	Durvalumab (an engineered human IgG1 mAb that blocks binding of programmed cell death ligand-1, PD-L1 to PD-1 and CD80) + Tremelimumab (selective human IgG2 mAb inhibitor of CTLA-4) vs. Durvalumab monotherapy vs. SOC (EXTREME regimen) CT in treatment naïve R/M HNSCC patients.	First-line treatment for R/M HNSCC targeting both PD-L1 and CTLA-4 pathways has potential for synergistic antitumor effects.<br>Exploratory endpoints include blinded independent central review of antitumor activity (immune-related RECIST v1.1) and potential biomarkers of progression/response.	Results ongoing
CONDOR (NCT02207530) Phase 2	Durvalumab + Tremelimumab vs. durvalumab monotherapy vs. Tremelimumab monotherapy in pts. with R/M HNSCC refractory to platinum-based therapy.	The PD-L1 and CTLA-4 pathways are non-redundant and preclinical data indicate targeting both may induce synergistic antitumor effects.<br>The primary endpoint was overall response rate (ORR) of D+T by blinded independent review committee using RECIST v1.1. Secondary endpoints included duration of response and overall survival (OS).	In combination, durvalumab monotherapy and Tremelimumab monotherapy cohorts, median OS: 7.6, 6.0 and 5.5 months; median PFS: 2.0, 1.9, 1.9; and ORR: 7.8, 9.2 and 1.6 %.<br>Both D and T monotherapy as well as the combination of D+T had acceptable toxicity in this pretreated, PD-L1 low/negative, R/M HNSCC population, and showed no new safety signals. D and D+T showed antitumor activity in a pt population with few treatment options. Phase 3 trials in first- and second-line HNSCC assessing D+T and D are ongoing with OS endpoints.
ICIs + innate immune activation			
KEYNOTE-184 (NCT02521870) Phase 1b/2	Intratumoural Injections of SD-101 (synthetic CpG-ODN agonist of TLR9) in Combination With Pembrolizumab in treatment-naïve R/M HNSCC patients.	SD-101 is a synthetic CpG-ODN agonist of TLR 9 that stimulates dendritic cells to release IFN-alpha and mature into antigen presenting cells to activate T cell anti-tumour responses.<br>Study DV3-MEL-01 (NCT02521870) assesses the safety and preliminary efficacy of SD-101 in combination with pembrolizumab in patients with recurrent or metastatic HNSCC.	Of the 10 evaluable patients, ORR by radiographic images was 33 %, compared to 15 % by pembrolizumab therapy alone.<br>In anti-PD-1 treatment naïve HNSCC patients, SD-101 in combination with Pembrolizumab showed a promising response rate and has been well tolerated. Full accrual of subjects in this cohort is ongoing.
STING (NCT02675439) Phase 1	STING (a stimulator of interferon genes) agonist MIW815 (ADU-S100) + Ipilimumab vs. Pembrolizumab in advanced solid tumours.	The STING pathway is a critical component of the antitumor response. ADU-S100 is a cyclic dinucleotide that activates all known human STING alleles.	STING promotes the growth of tumours characterized by low antigenicity via IDO activation.<br>Despite data demonstrating stimulation of the immune system by STING, the role of the STING pathway in anti-tumour immunity is still unclear.
ICIs + vaccine			
NCT02426892 Phase 2	Nivolumab + ISA101 (100 μg/peptide) in patients with incurable oropharyngeal cancer.	To determine if nivolumab efficacy is amplified through treatment with ISA 101, a synthetic long-peptide HPV-16 vaccine inducing HPV-specific T cells, in patients with incurable HPV-16-positive cancer.	Median PFS: 2.7 months and median OS: 17.5 months. Response was positively correlated with tumour cell PD-L1 positivity (≥1 %). 36 % ORR in patients with oropharyngeal cancer compared to 16 % by Nivolumab alone.<br>The overall response rate of 33 % and median overall survival of 17.5 months is promising compared with PD-1 inhibition alone in similar patients. A randomized clinical trial to

			confirm the contribution of HPV-16 vaccination to tumoricidal effects of PD-1 inhibition is warranted for further study.
NCT03162224<br>Phase 1b/2a	Safety and efficacy of MEDI0457 (a plasmid DNA vaccine, comprises 3 plasmids expressing HPV-16 and HPV-18 E6 and E7 proteins along with IL-12) + Durvalumab (a human IgG1 mAb that blocks programmed death-ligand 1, PD-L1 binding to PD-1, stimulating anti-tumour immune response) in patients with HPV+ R/M HNSCC.	Eligible patients include those with confirmed HPV+ HNSCC refractory to platinum-based CT.<br>Primary objectives are safety and objective response rate. Secondary objectives are disease control rate at 16 weeks, overall survival, progression-free survival, and pharmacokinetics and immunogenicity of durvalumab.	Currently recruiting.
ICIs + targeted therapy			
NCT02501096<br>Phase 1b/2	Lenvatinib (a multikinase inhibitor of vascular endothelial growth factor receptor 1–3, fibroblast growth factor receptor 1–4, platelet-derived growth factor receptor α, RET, and KIT) + Pembrolizumab combination therapy.	First systemic combination of a TKI and immunotherapy for patients with HNSCC.<br>The primary endpoint was ORR at 24 weeks. Secondary endpoints included ORR, progression-free survival (PFS), and duration of response (DOR), which is calculated for pts with complete or partial responses.	ORR at 24 weeks: 36.4 %. Grade 3/4 AEs occurred in 91 % of patients, with 18 % cases having to discontinue study treatment due to AEs.<br>LEN + PEM demonstrated promising clinical activity and manageable toxicities, supporting further evaluation of the LEN + PEM combination in pts with SCCHN.
Adjuvant therapy			
PATHWay<br>(NCT02841748)<br>Phase 2	Pembrolizumab vs. placebo in advanced HNSCC.	This randomized study is intended to explore the incorporation of Pembrolizumab into the treatment of patients with locally advanced HNSCC at high risk for recurrence or low-volume residual disease.	Currently recruiting. Eligible patients must have HNSCC, completed therapy with definitive intent, and have an estimated risk of recurrence ≥40–50 %.<br>The primary hypothesis of the study is that the addition of Pembrolizumab will improve the 2-year progression-free survival (PFS) rate, compared to placebo.
NRG HN-003<br>(NCT02775812)<br>Phase 1	Adjuvant Pembrolizumab + Cisplatin and IMRT in patients with high-risk, HPV-, stage III-IV HNSCC.	Giving pembrolizumab with cisplatin and IMRT to boost immune response.<br>Primary outcome is to determine the recommended phase II dose for the combination of Pembrolizumab and Cisplatin-radiotherapy in patients with high-risk, HPV- HNSCC, based upon dose-limiting toxicity.	Active.
NCT02892201<br>Phase 2	Pembrolizumab for patients with head and neck squamous cell carcinoma with residual disease following definitive chemoradiation.	Patients must be diagnosed with residual disease within 24 weeks of completion of radiation therapy. Residual disease must be biopsy proven before the patient can consent to the trial, and can be either from lymph nodes in the neck, or from the primary tumour site. Prior to beginning study therapy patients are evaluated by an ENT to determine if they have disease amenable to surgical resection. Both resectable and unresectable patients will be eligible for participation in the study.	Not recruiting. No results posted.
KEYNOTE-412<br>(NCT03040999)<br>Phase 3	Pembrolizumab + Chemoradiation and as maintenance therapy vs. Chemoradiation in pt with locally advanced HNSCC	The immune checkpoint inhibitor Pembrolizumab has previously demonstrated antitumor activity in recurrent and/or metastatic HNSCC in large Phase 3 trials. For patients with locally advanced disease, Phase 1b	Not recruiting.

		data on the use of Pembrolizumab in combination with chemoradiation have shown the approach to be safe and feasible.	
Neoadjuvant therapy			
KEYNOTE-689 (NCT03765918) Phase 3	Pembrolizumab as neoadjuvant therapy and in combination with standard of care as adjuvant therapy (SOC; radiotherapy ± cisplatin)	Dual primary end points are major pathological response, defined as ≤10% invasive squamous cell carcinoma within resected primary tumour and sampled regional lymph nodes per blinded central pathology, and event-free survival. Secondary end points include overall survival, pathological complete response, and safety and tolerability.	Recruitment is ongoing and will continue until ~600 patients are enrolled.
Checkmate-358 (NCT02488759) Phase 1/2	Neoadjuvant Nivolumab in patients with resectable HPV+ or HPV− HNSCC and EBV-associated NPC.	Treatment options for patients with R/M NPC are limited to palliative chemotherapy.<br>Primary endpoints were safety (incidence of TRAEs) and delay >4 weeks from planned surgical date due to TRAEs. Investigator-assessed change in tumor dimensions was an exploratory endpoint. Additional assessments include pathologic response, tumour PD-L1 expression, and immune correlates.	As of database lock, pre-surgery tumour reduction per CT scan was observed in 48 % evaluable pts. (≥40 % had tumour reduction, with largest reduction in 75 %).<br>Nivolumab was well tolerated, with no surgery delay due to TRAEs. Nivolumab led to tumor reductions within 1 month in nearly half of evaluable pts.
NCT02641093 Phase 2	Pembrolizumab in combination with SOC surgery followed by RT +/− cisplatin.	To test the ability of Pembrolizumab to improve locoregional recurrence and distant metastatic rates in high-risk patients with LA HNSCC treated with current SOC surgical approaches.	47 % of patients demonstrated a pathological response – high immune cell infiltration and amplified PD-L1 (>10 % tumor effect) and 32 % achieved a major response (> 70% tumour effect).<br>Pathological response was seen after one dose of pembrolizumab in HNSCC. Increased tumor immune cell infiltration predicted pathological response. Adjuvant combination treatment with pembrolizumab has an acceptable safety profile.

R/M HNSCCs: recurrent/metastatic head and neck cancers ICIs: Immune Checkpoint Inhibitors; CHT: Chemotherapy
